# Establishing a System for Testing Replication Inhibition of the *Vibrio cholerae* Secondary Chromosome in *Escherichia coli*

**DOI:** 10.3390/antibiotics7010003

**Published:** 2017-12-23

**Authors:** Nadine Schallopp, Sarah Milbredt, Theodor Sperlea, Franziska S. Kemter, Matthias Bruhn, Daniel Schindler, Torsten Waldminghaus

**Affiliations:** 1 LOEWE Center for Synthetic Microbiology-SYNMIKRO, Philipps-Universität Marburg, Marburg 35032, Germany; nadine@schallopp.de (N.S.); Sarah.Milbredt@ruhr-uni-bochum.de (S.M.); theodor.sperlea@staff.Uni-Marburg.DE (T.P.); Kemter@students.uni-marburg.de (F.S.K.); bruhnmatthias@aol.com (M.B.); daniel.schindler@manchester.ac.uk (D.S.); 2 School of Chemistry, Manchester Institute of Biotechnology, University of Manchester, Manchester M1 7DN, UK

**Keywords:** chromosome engineering, replication initiation, drug development

## Abstract

Regulators of DNA replication in bacteria are an attractive target for new antibiotics, as not only is replication essential for cell viability, but its underlying mechanisms also differ from those operating in eukaryotes. The genetic information of most bacteria is encoded on a single chromosome, but about 10% of species carry a split genome spanning multiple chromosomes. The best studied bacterium in this context is the human pathogen *Vibrio cholerae*, with a primary chromosome (Chr1) of 3 M bps, and a secondary one (Chr2) of about 1 M bps. Replication of Chr2 is under control of a unique mechanism, presenting a potential target in the development of *V. cholerae*-specific antibiotics. A common challenge in such endeavors is whether the effects of candidate chemicals can be focused on specific mechanisms, such as DNA replication. To test the specificity of antimicrobial substances independent of other features of the *V. cholerae* cell for the replication mechanism of the *V. cholerae* secondary chromosome, we establish the replication machinery in the heterologous *E. coli* system. We characterize an *E. coli* strain in which chromosomal replication is driven by the replication origin of *V. cholerae* Chr2. Surprisingly, the *E. coli ori2* strain was not inhibited by vibrepin, previously found to inhibit *ori2*-based replication.

## 1. Introduction

While the genetic setup in eukaryotic cells comprises multiple linear chromosomes, the standard in prokaryotes is a single circular chromosome [1]. The number of replication start sites is also different, with eukaryotic chromosomes starting replication at multiple origins, while all known bacterial chromosomes are replicated from a single origin of replication. However, in bacteria, some interesting exceptions occur in alternative genetic setups, including linear chromosomes and separation of the genetic information onto multiple chromosomes [2,3]. When two chromosomes exist in one bacterial cell, new questions arise about, for example, the timing of initiation and coordination of segregation in comparison to single-chromosome bacteria. The best studied two-chromosome bacterium is *Vibrio cholerae*, the causative agent of the cholera disease [4,5]. Chr1 of *V. cholerae* strain El Tor N16961 has a size of about 3 M bps and Chr2 of about 1 M bps [6]. Each of the two chromosomes has its own initiator protein to start replication at each single replication origin [7]; for Chr1, the initiator is DnaA, known to be the standard from studies in other model bacteria [8]. Meanwhile, the initiator for Chr2 is the protein RctB [8,9]. Although RctB is unique within the phylogenetic group of *Vibrionaceae*, it shows structural similarity to plasmid initiators [10,11]. This fits the common idea that Chr2 originates from a plasmid that was acquired by the cell early in evolution and then developed into a secondary chromosome [12]. One chromosome-like characteristic of Chr2 is its regulation in a cell-cycle dependent manner, attributed to the participation of SeqA and Dam methyltransferase in regulation of *ori2* [13,14,15]. Another feature of Chr2, further distinguishing it from plasmids, is that it encodes essential genes, although they are less frequent than when compared to the primary chromosome [16,17]. However, the plasmid ancestry of Chr2 is shown by the similarity of its replication initiation mechanism to plasmid systems; one such shared feature of both is the binding of the initiator protein to an array of specific binding sites, the so-called iterons [18]. In addition, handcuffing is involved in negative regulation of *ori2* as has also been shown for plasmid origins [12]. Although RctB alone is sufficient to melt the DNA double strand at *ori2*, this replication origin has been found to also depend on DnaA [8], the experimental evidence being the inability of the transfer of an *ori2*-based minichromosome to an *E. coli* strain lacking DnaA [5]. DnaA activity at *ori2* is probably linked to a conserved DnaA box some base pairs away from the iteron array [6,19]. Coordination of replication in the two-chromosome system of *V. cholerae* appears to work through the *crtS* site (=chromosome II replication triggering site), located on Chr1 and found to positively regulate initiation at *ori2* by binding RctB [20]. Regulation is thought to include physical contacts between *crtS* and *ori2*, as these two chromosome parts appeared to be coupled in chromatin conformation capture experiments [21]. Genetic changes of the *crtS* position on Chr1, either closer to *ori1* or further away, resulted in a corresponding shift of Chr2 initiation time as seen by a changed copy number [21], showing that the native position of the *crtS* sets the timing of Chr2 replication to finish in synchrony with Chr1 replication [21,22,23]. An important tool in studies on *V. cholerae* DNA replication was and is the use of *E. coli* as a heterologous host. First evidence of which sequences function as replication origins in *V. cholerae* came from testing their ability to replicate a corresponding minichromosome in *E. coli* [5]. Later, the native replication origin of *E. coli* was replaced by the very similar *ori1* of the primary *V. cholerae* chromosome [13,14], which was used to show that the Dam methyltransferase is not essential for *ori1* replication and can thus not be responsible for Dam being essential in *V. cholerae* (and not in *E. coli*). The conclusion was therefore that Dam-dependent methylation of *ori2* is crucial for initiation of replication; this assumption was confirmed by showing firstly that RctB binding sites need to be methylated in order to be bound by RctB, and secondly, that Dam loss selects for chromosome fusion in *V. cholerae*, omitting the need for a functional *ori2* since all genetic material can be replicated by *ori1* [13,24]. Attempts to construct an *E. coli* strain with *V. cholerae ori2* driving DNA replication instead of *oriC* as an important tool to study related questions had previously been unsuccessful [13]. Here, we study replication of such a strain with an insertion of *ori2* including the genes encoding *parAB* and *rctB* at position 4,422,941 of the *E. coli* chromosome and an *oriC* deletion that was constructed on the way toward developing an *E. coli* strain with two chromosomes [25]. We show that chromosomes over-replicate in *E. coli* with an *ori2* origin and that its replication is indeed dependent on Dam. Further experiments assess the relationship of *crtS* to *ori2* copy number, the role of DnaA in *ori2* initiation and make use of the genetic system to study a chemical compound that was described to act specifically on RctB. Finally, we show that *ori2* can replace *oriC* at its native location by constructing a corresponding strain.

## 2. Results

### 2.1. V. Cholerae ori2 Dependent Replication of the E. coli Chromosome

**Figure 1:**
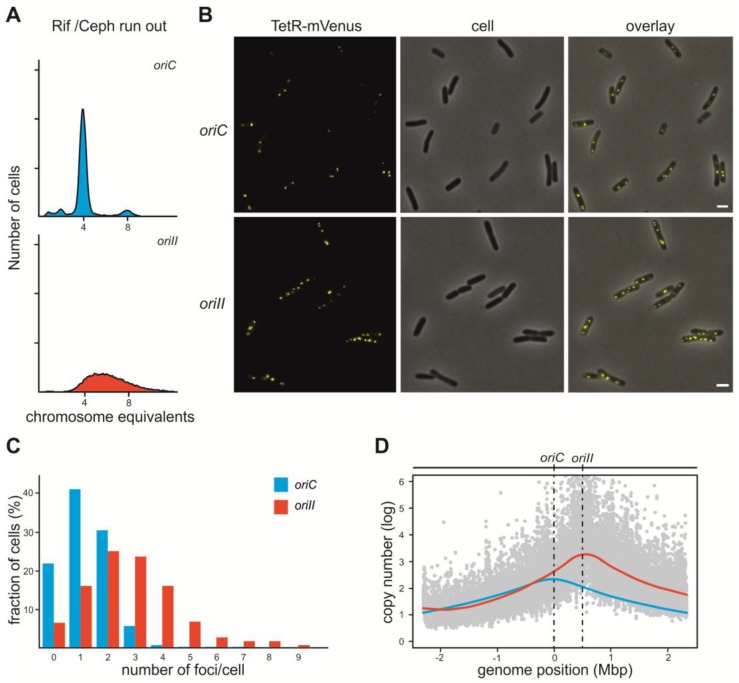
DNA replication in *E. coli* strains with *oriC* or *ori2* driven replication. (**A**) Flow cytometry analyses of DNA content in rifampicin/cephalexin treated *E. coli* cells with DNA replication starting at *oriC* (strain #1; top panel) or *V. cholerae ori2* (strain #16; bottom panel) [25]. (**B**) Fluorescence microscopy of *E. coli* cells harboring a plasmid encoding a TetR-mVenus fusion (pMA289), a FROS array insertion and either *oriC* (top panel; strain SM112) or *ori2* (bottom panel; strain SM113). The scale bar is 2 µm. (**C**) Quantification of fluorescence foci per cell for microscopy shown in B (n = 700). (**D**) Profile of genome-wide copy numbers based on comparative genomic hybridization (CGH). Grey dots represent values of single probes for the *ori2*-based strain (#16) with a Loess regression (red line, F = 0.2). For comparison, the Loess regression of the *oriC*-based strain #1 is shown based on published data [29] (blue line, F = 0.2). Positions of *oriC* and *ori2* are indicated and the genomic position as distance from *oriC*.

In order to establish a test system for replication inhibitors of *V. cholerae ori2* we analyzed an *E. coli* strain which has this origin including the flanking genes *rctB* and *parAB* inserted at an ectopic site and the native *oriC* being deleted (strain #16) [25]. Exponentially growing cultures were treated with rifampicin, which blocks replication initiation, and cephalexin, which inhibits cell division [26]. In wildtype *E. coli* (wt) cells, this treatment led to cells containing either 4 or 8 fully replicated chromosomes (Figure 1A). However, the flow cytometry histogram of a strain in which chromosomal replication is driven solely by *ori2* (strain #16) looks completely different (Figure 1A). Total DNA content in the strain driven by *ori2* is higher on average than when compared to the wt strain, and no distinct peaks are visible indicating unfinished replication rounds. Our analysis fits a chromosome replicated by *ori2* in this strain, as *ori2* was shown to be insensitive to rifampicin treatment in comparison to *oriC* in *E. coli* and *ori1 in V. cholerae* [22,23]. Clearly, a different experimental approach is needed to assess if *ori2*-driven replication in *E. coli* has similar timing to *oriC*-based replication, or if initiation timing is different and/or potentially disturbed. To answer this question, we used quantitative fluorescence microscopy based on a recently constructed FROS array inserted into the *lacZ* locus [27] (Figure 1B,C). Since the FROS array marks one region of the chromosome, the number of respective foci should correlate with chromosome copy numbers. In exponentially growing cells of the *ori2*-strain (SM113), we could detect a clear increase in the number of foci compared to the control strain with *oriC* (SM112) (Figure 1B,C). This observation suggests that the *ori2*-based chromosome in *E. coli* over-initiates, in comparison to the native *oriC*-driven replication. To verify this finding, we performed microarray-based comparative genomic hybridization (CGH) to examine genome wide gene copy number patterns (Figure 1D). Over-initiation should lead to an increased origin to terminus copy number [28]; indeed, the strain with *ori2* had an origin copy number of 3.2, which is significantly more than the wt strain under similar growth conditions (Compare red and blue line in Figure 1D). These results indicate that replication of the primary *E. coli* chromosome by *ori2* increases the number of initiation events within one cell cycle in comparison to wt *E. coli*. The CGH experiments also confirmed *ori2* to be the only active replication origin in the analyzed strain because the copy number maximum appeared at the chromosomal position of *ori2* insertion (Figure 1D).

### 2.2. Initiation of Replication at ori2 in E. coli Depends on Dam Methylation

**Figure 2:**
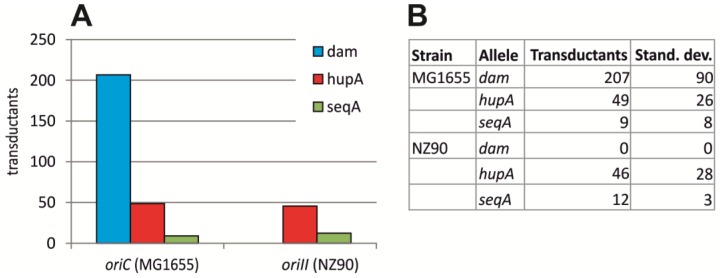
Transduction efficiency of gene knockouts into *oriC* and *ori2 E. coli* strains. Colonies growing on plates supplemented with kanamycin were counted after P1 transduction of KanR-cassettes inserted in *dam*, *hapA* or *seqA* as indicated into *E. coli* strains, with chromosome replication based on *oriC* (MG1655 (#1)) or *ori2* (NZ90). Mean values of three replicates are shown in (**A**) with the actual numbers and standard deviation given in the table (**B**).

Dam methyltransferase was shown to be essential in *V. cholerae* [30]. An *E. coli* strain with *oriC* substituted by *V. cholerae ori1* was instrumental to show that this is due to effects of Dam on *ori2* and not *ori1*, as Dam was not essential in this strain [13,14]. Therefore, one conclusion would be that Dam should be essential in an *E. coli* strain with *ori2* driving chromosomal replication. To test this hypothesis, we analyzed the transfer of a Δ*dam* allele by P1 transduction to an *E. coli* strain in which replication is solely driven by *ori2* (#16). While the deletion could easily be introduced to *E. coli* wt cells, no transduction was found in strain #16 (Figure 2). In contrast, the positive control *hupA*, encoding one subunit of the DNA-binding protein HU, could be transduced into both strains with similar efficiencies (Figure 2). The same was observed for an antibiotic cassette insertion within the *seqA* locus. This result confirmed previous findings of SeqA not being essential for *ori2*-dependent initiation of DNA replication.

### 2.3. crtS-Dependent Regulation of ori2-Based Replication in E. coli

**Figure 3:**
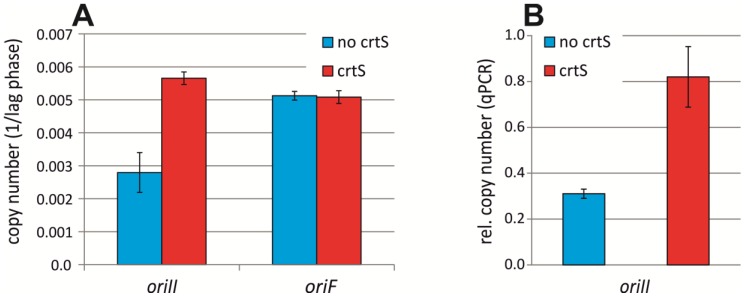
Copy numbers of secondary replicons in *E. coli* dependent on *crtS*. (**A**) Copy number of an *ori2*-based minichromosome in an *E. coli* strain without *crtS* (Strain NZ72) or with *crtS*-site insertion on the chromosome (Strain NZ140) was measured as inverse of the lag time for cells in medium with high concentrations of ampicillin (500 µg/ mL). Strains carrying an *oriF*-based replicon and the *crtS* (Strain NZ141) or no *crtS* on the chromosome (Strain NZ119) were used as control. Data are the mean of three biological replicates with the indicated standard deviations. (**B**) Copy number of an *ori2*-minichromosome (pMA568) analyzed by qPCR-based marker frequency analysis relative to *oriC*. Mean values of three biological replicates are shown with the respective standard deviations.

*V. cholerae* Chr1 was found to encode a short DNA sequence called *crtS* (*chromosome II replication triggering site*) that regulates initiation at *ori2* [20,21]. We hypothesized that the *ori2*-driven chromosome in strain #16 is dysregulated due to lacking a *crtS* site. The *crtS* site was found to increase the copy number of an *ori2*-minichromosome in *E. coli* [20]. However, in those experiments, *crtS* was supplied in multiple copies through a pBR322 plasmid, which is unlike the situation in *V. cholerae*. To analyze the effect of more physiological numbers of *crtS* on *ori2* replication, we inserted a *crtS* site on the *E. coli* wt chromosome driven by *oriC* between genes *fucR* and *rlmM* (genomic position ~2,940,120) about 1 M bps from *oriC*. The strain was transformed with an *ori2*-minichromosome and its copy number determined via quantification of the sensitivity towards elevated concentrations of antibiotics, the underlying logic being that higher replicon numbers lead to higher gene dosage of the encoded resistance gene and correspondingly to a higher tolerance towards respective antibiotics [31,32,33]. An increased copy number of the *ori2*-minichromosome was observed in a strain carrying the *crtS* insertion, compared to the wt lacking *crtS* (Figure 3A). Notably, this effect was *ori2* specific, since a replicon driven by the F-plasmid replication origin did not show differential copy numbers (Figure 3A). To verify these results, we measured *ori2*-minichromosome copy numbers relative to the primary *E. coli* chromosome by qPCR (Figure 3B). We found an increased copy number of the *ori2*-minichromosome from 0.31 ± 0.02 in wt to 0.82 ± 0.13 in the strain with a chromosomal insertion of *crtS*. Our data confirmed that *crtS* increases the *ori2*-minichromosome copy number and showed that a chromosomal *crtS* insertion is sufficient to this end.

**Figure 4:**
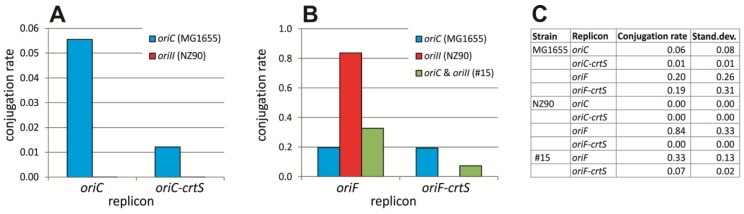
Conjugation rate of extra replicons to *oriC* and *ori2 E. coli* strains. (**A**) Transfer by conjugation was tested for *oriC*-minichromosomes carrying a *crtS* site (pMA200) or no *crtS* (pMA308) into *E. coli* with chromosome replication based on *oriC* (MG1655) or *ori2* (NZ90). (**B**) Conjugation of *oriF*-based replicons with *crtS* (pMA206) or without (pMA899) into *E. coli* with *oriC* (MG1655), *ori2* (NZ90) or *oriC* at the native site and *ori2* at an ectopic site (#15) [25,29]. Mean values of three biological replicates are shown in (**A**,**B**) with the actual numbers and standard deviations given in the table (**C**).

We speculated that a *crtS* site could lead to more regular replication timing of the primary chromosome in the *E. coli* strain #16 with *ori2*-driven chromosome replication. To make *crtS* replication dependent on the cell cycle, as is the case in *V. cholerae*, we constructed an *oriC*-minichromosome carrying a *crtS* site. However, transformation of strain #16 failed by this replicon failed. To study this phenomenon in detail we quantified the number of colonies as a result of conjugating the replicon from a donor strain to either an *E. coli* wt, or strain #16 with *ori2*-driven chromosomal replication. An *oriC*-minichromosome was efficiently transferred into a wt *E. coli* by conjugation, as was an *oriC*-minichromosome carrying a *crtS* site (Figure 4A). In contrast, neither of these two replicons could be transferred to strain #16 via conjugation. These results indicated that it is the *oriC* on the extra replicon which causes some problem in the *ori2 E. coli* strain. As an alternative to test the effect of *crtS* on chromosomal *ori2* replication, we constructed a replicon with the F-plasmid origin and a *crtS* site. Interestingly, this replicon could also not be transferred to strain #16 (Figure 4B). One explanation for lacking transconjugants could be that the *crtS* site leads to repression of the extra replicon for example by strong binding of RctB. An alternative explanation would be that the *crtS* site effects *ori2* replication on the primary chromosome in a way leading to cell death. To distinguish between these two possibilities we tested conjugation into a strain carrying *oriC* and *ori2* on the primary *E. coli* chromosome (Figure 4B). If replication problems would be due to events on the extra replicon it should not be possible to conjugate into this strain. However, transconjugants were observed, indicating that some interference of the *crtS* site with *ori2*-based replication on the *E. coli* chromosome hinders extra replicons carrying a *crtS* site to be transferred to strain #16.

### 2.4. Assaying the DnaA-Box in ori2 for Its Role in Replication Initiation

**Figure 5:**
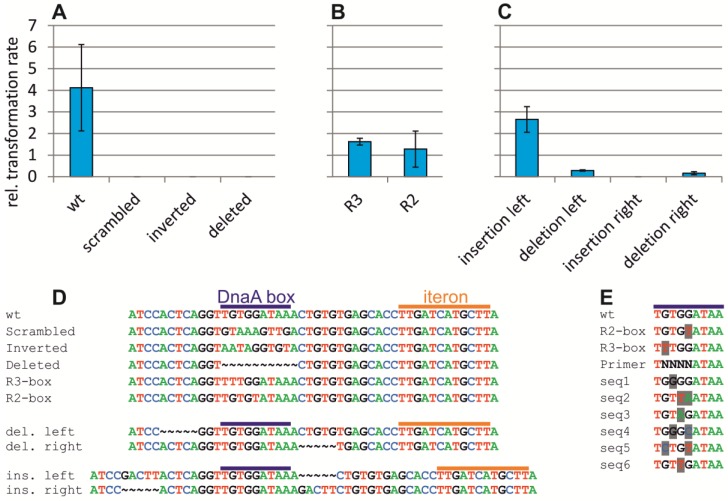
Mutation-based analysis of the DnaA box within *ori2.* (**A**–**C**) *Ori2*-based minichromosomes with different mutations at the DnaA box were tested for their ability to replicate by transformation into *E. coli* (XL1-Blue) or DH5αλ*pir*. The latter was used as a control because all replicons carry an oriR6K which allows replication in strains encoding the initiator Pir. Values are ratios between respective colony numbers of three biological replicates with the indicated standard deviations. Relevant sequences are shown in (**D**) for DnaA boxes wt (pMA87), scrambled (pMA108), inverted (pMA109), deleted (pMA110), a weak DnaA box R3 (pMA111), a medium-strength DnaA box R2 (pMA112), a 5 bp insertion to the left of the DnaA box (pMA115) or the right (pMA114) or a 5 bp deletion to the left (pMA113) or right (pMA116) as indicated. (**E**) Sequences found by transformation of an assembled *ori2*-minichromosome with a mix of sequence combinations at positions 2–5 as indicated by “N” in the Primer sequence. DnaA-box sequences from *oriC* in *E. coli* are shown for comparison (wt, R2, R3). Six sequences found in the screen are shown with the nucleotides differing from the consensus DnaA box shaded in grey.

Our observation that an *oriC*-minichromosome could not be transferred to an *E. coli* strain with *ori2*-based chromosome replication suggested a mechanism of interference. Such an effect has been observed in cases where the two origins differ in their efficiency to initiate replication [13,34]. However, such interference is usually due to the competition or interference of factors used by both replication origins. One factor that both *ori2* and *oriC* require for replication is DnaA. While the role of DnaA as initiator at *oriC* and *ori1* is well studied, much less is known about DnaA activity at *ori2*. It has been established that *ori2*-dependent replication requires DnaA and that *ori2* contains a DnaA box beside the RctB binding iterons, which appeared in a mutagenesis screen to be functionally important [5,19]. The role of the DnaA box was initially tested by the introduction of some mutations into a minichromosome carrying the minimal *ori2* sequence [19]. Here, we constructed and tested some of these mutations as well as additional ones in a minichromosome system including both the entire *ori2* and the flanking *rctB* and *parAB* genes. Functionality of minichromosomes was tested by measuring their transformation efficiency of a wt *E. coli*. This efficiency was measured relative to transformation of the same replicon into a strain encoding the λpir gene, which allowed replication based on the *oriR6K* of the *ori2*-minichromosomes. Deletion, inversion or scrambling of the DnaA-box rendered *ori2* nonfunctional, as seen by the inability of the respective replicons to transform a wt *E. coli* (Figure 5A). How well a DnaA box matches the consensus sequence determines the affinity of DnaA towards this site. Since the DnaA box within *ori2* has the sequence of a high-affinity binding site, we tested the effect of changing this sequence to a weak or medium-strength DnaA box (Figure 5B). Both alternative DnaA boxes allowed *ori2*-minichromosome replication; however, the transformation efficiency was reduced, indicating that a high affinity DnaA box is needed to allow optimal functioning of *ori2*. The distance between the DnaA box and the first of six RctB binding iterons is 22 bps, placing the two proteins on the same face of the DNA helix. We tested the insertion or deletion of 5 bps between these sites which should shift the binding sites half of a DNA turn along (Figure 5C). Insertion and deletion to the right of the DnaA box (between DnaA box and iteron) greatly reduced the transformation efficiency in comparison to wt or to an insertion to the left. This finding indicated that the distance between the DnaA box and the iterons is critical for its functionality. Interestingly, a deletion to the left of the DnaA box also reduced the transformation efficiency.

As an alternative approach to assess the importance of the DnaA box for *ori2* functionality, we cloned the *ori2* fragment into a plasmid based on a sequence library that substituted four nucleotides of the DnaA box with all possible 256 sequence combinations (Figure 5D). Transformation of the respective minichromosome mix into *E. coli* wt should then allow selection of functional replication origins. Sequencing of *ori2* sequences derived from such experiments revealed six different sequences that could function in place of the original DnaA box (Figure 5D). In all sequences found, two or three out of four nucleotides matched the DnaA box consensus, confirming the importance of the DnaA box on one hand and some potential for variation on the other, supporting the above results on introduction of weaker DnaA boxes.

### 2.5. Vibrepin Does Not Inhibit ori2-Dependent Replication

**Figure 6:**
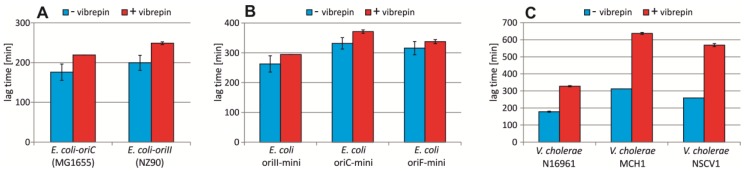
Effect of vibrepin on *ori2*-dependent replication. Lag time (time until OD = 0.1 was reached) is shown as the mean value of three replicates, with the respective standard deviations, in medium without vibrepin (blue) or with vibrepin (red). Vibrepin concentrations were 16 μg/mL for *E. coli* strains (**A**–**B**) and 1.6 μg/mL for *V. cholerae* strains (**C**). Analyzed *E. coli* strains replicated their chromosome based on *oriC* (MG1655) or *ori1* (NZ90) (**A**) or carried an extra minireplicon with *ori2* (pMA100), *oriC* (pMA106) or *oriF* (pMA129) [38] (**B**) as indicated. *V. cholerae* strains are the standard two-chromosome strain N16961 [6], an engineered derivative of N16961 with fused chromosomes (MCH1) [36] or a natural isolate with fused chromosomes (NSCV1) [37].

A screen for substances that lead to growth inhibition of an *E. coli* strain carrying an *ori2* minichromosome uncovered such activity of 3-(3,4-dichlorophenyl)cyclopropane-1,1,2,2-tetracarbonitrile, designated as “vibrepin” (for *Vibrio* replication inhibitor) [35]. It was found that vibrepin interferes with *ori2*-opening activity of RctB [35]. We reasoned that vibrepin should inhibit growth of *E. coli* strain #16 with chromosomal replication solely based on *ori2*, and as such the strain might be useful to identify additional *ori2*-specific inhibitors in the future in order to derive *V. cholerae*-selective antibiotics. Comparing the lag-phase duration of an *E. coli* wt with and without vibrepin revealed a slightly longer lag phase (13.5%) in medium supplemented with 16 µg/mL vibrepin (Figure 6A). Surprisingly, this difference was similar in the *ori2* strain (Figure 6A) indicating that vibrepin does not significantly inhibit *ori2* initiation in this context. As a second line of evidence, we compared the effect of vibrepin on *E. coli* cells carrying extra replicons based on either *ori2*, *oriC* or the F-plasmid origin (Figure 6B). The elongation of lag phases caused by vibrepin was similar for all three replicons, confirming the above results that vibrepin was not acting on *ori2* specifically (Figure 6B). To test vibrepin activity on *ori2* in *V. cholerae* directly, we compared its effect on growth of (i) strain N16961 carrying a secondary *ori2*-based chromosome [6], (ii) strain MCH1 with a fused chromosome driven by *ori1* and lacking *ori2* [36], and (iii) the recently characterized *V. cholerae* strain NSCVI with fused chromosomes and intact copies of *ori1* and *ori2* [37]. If vibrepin acts against *ori2* specifically, one would expect strain N16961 to be inhibited but not the two other strains. However, we found vibrepin to have an effect on the lag phase duration for all three strains, indicating that vibrepin inhibits *V. cholerae* growth, but not based on interference at *ori2* (Figure 6C).

### 2.6. ori2 Can Replace oriC in E. coli

**Figure 7:**
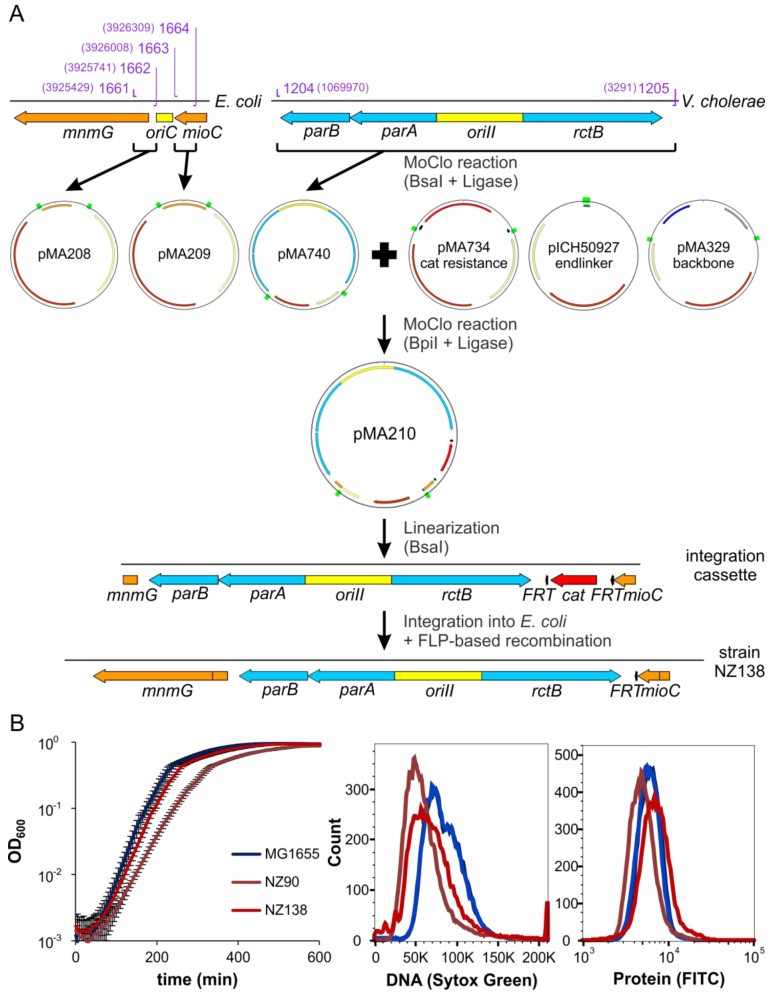
Construction and characterization of strain NZ138. (**A**) Scheme of NZ138 construction. Genes are indicated by arrows, origins of replication and the truncated end of *mnmG* by blocks. Genes of *V. cholerae* are colored blue, genes of *E. coli* orange, resistance genes red, FRT-sites black and origins of replication yellow. Green rectangles indicate BsaI and BpiI restriction sites. Binding sites of oligonucleotides are indicated in purple, numbers in brackets are genome positions of 5’-ends of the binding sites. Genomic and linearized DNA is indicated by grey lines, plasmids by circles. (**B**) Growth, DNA content and protein content of NZ138 compared to MG1655 and NZ90. All strains were grown in LB medium. For growth curves, five replicates for each strain were grown in a 96-well plate at 37 °C. For determination of DNA and protein content, samples were taken in exponential phase and fixed with ethanol. The samples were split, stained with SYTOX Green (DNA) or FITC (protein) and analyzed by flow cytometry.

We have characterized here an *E. coli* strain with an ectopic *ori2* insertion about 500 kbps away from *oriC* combined with a deletion of *oriC* and used it to study biological features related to *ori2*-based replication. The value of such a system was appreciated before but attempts to replace *oriC* by *ori2* were unsuccessful [13]. We reasoned that such an exchange of *oriC* to *ori2* should be possible in general based on the findings outlined above. Indeed, we were able to replace the *oriC* sequence in *E. coli* strain MG1655 with the full *V. cholerae ori2* sequence, including the flanking genes *rctB* and *parAB* (Strain NZ138, see Figure 7A and Methods section for details). Successful *oriC* replacement was confirmed by the inability of a Δ*dam* allele to be transferred by P1 transduction to the constructed strain, similar to what was found for strain #16 above. A comparison of a wt *oriC*-strain, to this new *oriC* to *ori2* exchange strain (NZ138) revealed a slight increase of doubling time (21.6 vs. 22.6 respectively; Figure 7B). The strain with the ectopic *ori2* insertion (NZ90) grew slower (27 min doubling time). DNA contents, as measured by flow cytometry were lower for both *ori2* strains compared to the *oriC* wt strain (Figure 7B, middle panel). The protein content as measure of the cell size varied slightly with cells of strain NZ90 being smallest on average and NZ138 being the largest cells (Figure 7B, right panel). In summary, characteristics of the newly constructed stain NZ138 differ from those of strain NZ90 and are more similar to a wt *E. coli* with *oriC*-driven replication. We believe that this *E. coli ori2* strain NZ138 will be of great help to derive new insights into *ori2*-dependent replication and screen for new *V. cholerae* specific replication inhibitors in the future.

## 3. Discussion

DNA replication is an attractive target for the development of antimicrobials. However, engineered systems are needed to be able to search specifically for chemicals targeted against the replication mechanism of interest. Such engineering approaches have for example used expression of hyperactive mutant proteins that kill the cells unless a chemical component suppresses their action [39]. The replication system of the secondary *V. cholerae* chromosome is especially interesting in respect to the development of antimicrobials because it is unique in the Vibrionaceae. The replication origin *ori2* can be studied in *E. coli* regarding general functionality and drug development using so-called minichromosomes [5,35]. These are plasmid-like replicons carrying a chromosomal replication origin and usually a conditional plasmid origin to be able to construct the replicons in the first place [40]. Minichromosomes are an important tool to, for example, identify the minimal region of a chromosome that functions as its origin of replication. Due to their easier genetic manipulation, they are also used frequently to characterize replication origins further—for example, in identification of the role of individual DNA motifs, as demonstrated with the DnaA box in *ori2* above (Figure 5). However, minichromosome-based studies also have drawbacks in both their small size and their competition with the chromosomal replication origin. *OriC*-minichromosomes have high copy numbers due to a lack of an equipartition mechanism [41]. The competition can lead to integration of minichromosomes into the primary chromosome [42]. An alternative to minichromosomes is the substitution of native chromosomal replication origins with the *ori* of interest. For instance, such an approach was instrumental in order to study the role of Dam methyltransferase and SeqA for *V. cholerae ori1* based replication [13,14]. However, while it is easy to see if a certain mutation is rendering a replication origin non-functional when using minichromosomes, investigating replication origins driving replication of the primary chromosome is not straightforward, as replication here is an essential process. One solution to this problem is the insertion of an additional plasmid origin which can be switched off, by, for example, repression of its initiator protein [43]. If depletion of an inducer leads to cell death, the origin of interest must be non-functional [43]. An alternative to this approach is the analysis of chromosomes carrying two functional replication origins which, for example, allow conclusions on their replication timing to be drawn by comparison with their MFA profiles [29]. The study showed that *E. coli oriC* and *V. cholerae ori2* can coexist on the chromosome and both be active [29]. This is consistent with many studies using *ori2*-based minichromosomes to study *V. cholerae* replication in *E. coli*. It was thus surprising that an *oriC*-minichromosome could not be transferred to an *E. coli* strain with chromosomal replication based on *V. cholerae ori2* (Figure 4A). We propose three possible reasons to explain this phenomenon; (i) the *oriC*-minichromosome leads to *ori2*-based over-replication of the chromosome, (ii) the *oriC*-minichromosome leads to *ori2*-based under-replication of the chromosome, and (iii) the interference of *oriC* and *ori2* hinders *oriC*-dependent minichromosome replication. Regarding the first possibility, over-replication is a commonly observed phenomenon caused by different mutations that might be tolerated in some cases and leading to cell death in others [44,45,46,47]. The differentiation between the other two hypotheses is related to the question of which origin can compete better for factors needed for replication initiation at both *oriC* and *ori2*. Three known protein factors are relevant in this context; SeqA, Dam and DnaA. SeqA is a negative regulator of initiation at both replication origins and competition should therefore lead to more initiations but never to a blocking of replication [13,48,49]. The role of Dam is to methylate the GATCs which occur at both replication origins, but the speed of the methylation process is so fast that a changed *ori2* methylation due to an increased number of GATCs on the *oriC* minichromosome is unlikely. These considerations leave DnaA remaining as the factor required by both *ori2* and *oriC*. It is well conceivable that *oriC* would win a competition for DnaA against *ori2* since it comprises multiple DnaA binding sites, while *ori2* has only a single one. Even more DnaA is directed away from *ori2* when the *oriC* on the minichromosome occurs in multiple copies [38,41]. A single *oriC* copy on the chromosome could also inhibit *ori2*-driven replication initiation, but perhaps only to a minor extent. Interestingly, this seems to be the case in practice, as copy numbers of *ori2* in *E. coli* strains that carry the native *oriC* are lower than *oriC* or similar. This is the case for *ori2* minichromosomes as well as ectopic *ori2* insertions [29,33,38]. This is remarkable, since *ori2* itself was shown to lead to over-initiation and higher chromosomal copy numbers without a competing *oriC* in the cell (Figure 1). These findings suggested that the availability of DnaA limits initiation at *ori2*. However, replication of Chr2 in *V. cholerae* did not increase with over-expression of DnaA while Chr1 copies were amplified [7]. One possible explanation for this discrepancy is that it is not the amount of DnaA per se that is important, but the availability of ATP vs. ADP-bound versions of this protein. Our data seem to suggest that DnaA-ATP is actually functional at *ori2*, based on our finding that lower affinity DnaA boxes can replace the native DnaA box (Figure 5B,E). These sites are able to bind ATP-DnaA but not ADP-DnaA [50]. The exact role of DnaA in replication initiation at *ori2* remains to be uncovered but mutational analyses of *ori2*-based minichromosomes presented here and in a previous study provide first insights (Figure 5) [19].

It appears that DnaA may add just another layer of regulation to the regulation of Chr2 replication in *V. cholerae* alongside SeqA, Dam methylation, and the recently found *crtS* site. The latter was found here to increase the copy number of an *ori2*-minichromosome when it was itself located on the *E. coli* chromosome (Figure 3). If a slight increase in the number of initiation events at *ori2* is the general effect of *crtS*, it is hard to find an explanation for the result where an F-plasmid-origin-based replicon carrying a *crtS* site could not be transformed into the *ori2 E. coli* strain (Figure 4). Notably, we were also not able to transfer the chromosomal *crtS* insertion into the *ori2*-strain. We suggest that *crtS* and *ori2* are actually regulated in a way that adjusts their copy numbers to be similar. This is what was found in *V. cholerae*, where copy numbers of Chr2 increased when the *crtS* was moved closer to *ori1*, coinciding with an increased copy number of *crtS*. Comparison to previous studies on *crtS* in *E. coli* are difficult because they used a three plasmid system with *ori2*, *rctB* and *crtS* each on an extra replicon [20]. Certainly, further research is needed to fully understand the mechanistic *crtS*-to-*ori2* interrelation. Nevertheless, we consider the heterologous *E. coli* system with chromosomal insertions of *ori2* and *crtS* presented here to be highly valuable in the study of the complex replication regulation system of *V. cholerae* in a simplified synthetic system. *Ori2* was used previously to search for replication inhibitors specific for *V. cholerae* and we anticipate that corresponding studies might be extended to the *crtS* site mechanism potentially with the experimental system presented here and its further development [35].

We have used the *ori2*-test system established here to investigate the activity of the chemical compound 3-(3,4-dichlorophenyl)cyclopropane-1,1,2,2-tetracarbonitrile, designated as “vibrepin” (for *Vibrio* replication inhibitor). Our results confirm the inhibition of *V. cholerae* growth that was found previously (Figure 6C) [35]. Surprisingly, vibrepin did not inhibit growth of *E. coli* strain NZ90 with *ori2*-driven chromosome replication more than that of a wt *E. coli* strain with *oriC*-based replication. This finding contradicts previous results showing that RctB is specifically inhibited by vibrepin both in vivo and in vitro [35]. This conclusion was also based on a plasmid-based *ori2*-system in *E. coli* similar to our minichromosome approach (Figure 6B) [35]. However, one difference of our replicon to the previously used one is the inclusion of the *parAB2* operon, encoding the partitioning proteins. Also, the *E. coli* strains with *ori2*-driven replication of the primary chromosome studied here, encode the *parAB2* operon. Notably, ParB2 was shown to participate in regulation of initiation at *ori2* [51]. One possibility to explain the contradicting results might thus be linked to the role of ParB2 in DNA replication initiation. It could, for example, be that ParB2 counteracts the activity of vibrepin against RctB in a yet unknown fashion yet to be discovered. Interestingly, the previous study suggested that vibrepin might have other targets in addition to RctB within *V. cholerae*. This note supports the need for heterologous systems as established in the project presented here to be able to determine specificities of inhibitors.

## 4. Materials and Methods

### 4.1. Bacterial Strains, Plasmids, Oligonucleotides, and Culture Conditions

Strains, plasmids and oligonucleotides are listed in Table S1–S3. Pre-cultures of *E. coli* were grown in 3 mL LB medium. Antibiotics and inducer were used with the following concentrations if not indicated otherwise: ampicillin (100 μg/mL), kanamycin (35 μg/mL), chloramphenicol (35 µg/mL). Growth curves were measured in 96-well plates in a microplate reader (Victor X3 Multilabel plate reader, PerkinElmer or Infinite M200pro multimode microplate reader, Tecan, Männedorf, Switzerland) at 37 °C. The 150 µL of main culture was inoculated 1:1000 and covered with 70 µL of mineral oil. Optical density was measured in 5 or 6-minute intervals.

### 4.2. P1 Transduction

P1*vir* transduction was carried out as described [52]. Equal cell numbers of strains #1 and NZ90 were incubated with the same P1*vir* lysate diluted 1:10 in steps to 10^−3^. Cells were plated after a 1 h recovery, plated on selective medium and incubated overnight. Colonies resulting from transduction with P1*vir* lysate dilution of 10^−2^ were considered for all experiments with colony numbers ranging from 0 to 300.

### 4.3. Conjugation

The donor and acceptor strains were grown overnight and cell numbers adjusted to OD_600_ 3.0. 100 µL of cells were washed three times in LB medium supplemented with 300 µM DAP to remove antibiotics. 50 µL of both cell suspensions were mixed and 50 µL of the mix spotted on an LB-agar plate containing DAP. Conjugations were incubated for 5 h at 37 °C and about 1/3 of the drop scratched off the plate and re-suspended in 1 mL LB medium. Cells were washed three times in 1 mL LB and finally re-suspended in 100 µL of dilution buffer. Suspensions were diluted stepwise 1:10 until 10^−9^ and 20 µL of each dilution plated on LB with selection for the transconjugants (LB-amp without DAP) and the acceptor cells (LB without DAP), respectively. Plates with colony numbers suitable for counting were selected after overnight incubation and the ratio between transconjugants and acceptor calculated as measure of conjugation rate.

### 4.4. Quantification of Replicon Copy Numbers by qPCR

In exponential growth phase, 1 mL culture of the *E. coli* strains NZ72 and NZ140 grown in LB-amp was harvested by centrifugation at 15,000× *g* for 4 min. The cells were stored at –20 °C. After thawing on ice, the sample were re-suspended in 1 mL water and incubated at 95 °C for 10 min. According to the formula OD_600_ 1 = 8 × 10^5^ cells/μL, the samples were diluted to 1.25 × 10^4^ cells/μL. The *E. coli* strain FSK18 was used as reference. It was grown in LB to early exponential growth phase and incubated with 150 µg/mL rifampicin and 10 µg/mL cephalexin at 37 °C for 3.5 h. Each reaction was carried out in triplicates of 10 μL. Primer sets for *oriC* (3921366fw, 3921366rv, 3921366pr) and *synVicII* (ori2fw, ori2rv, ori2probe) were used in separate reactions. Three biological replicates were analyzed three times each. Ratios of *synVicII* to *oriC* were calculated relative to strain FSK18 containing the genomic regions that are template for the *oriC* and *synVicII* primer sets (see above). qPCR reactions were composed of 5 μL KAPA Probe Fast qPCR mastermix Universal 2× (peqlab, Erlangen, Germany), 1 μL primer mix and 4 μL cell suspension. The 250 μL primer mix was prepared for each set of primer and contains 22.5 μL primer fw (100 pmol/μL), 22.5 μL primer rv (100 pmol/μL), 6.25 μL probe (5′-Fam/3′-Tamra, 100 pmol/μL), 50 μL Rox Reference Dye Low 50× (peqlab) and 148.75 μL water. qPCR reactions were performed in the real-time cycler qTower (Analytik Jena AG, Jena, Germany) with the following program: 1, 95 °C for 3 min; 2, 95 °C for 3 s; 3, 55 °C for 20 s; and 4, fluorescence read. Steps 2–4 were repeated 45 times. The determination of the CT-values was carried out with the software qPCRsoft (Analytik Jena AG, Jena, Germany) without using the rox reference.

### 4.5. Quantification of Replicon Copy Number via Antibiotic Sensitivity

Analysis of the copy-up effect of *crtS* was done as described in [33]. Cells were grown in LB medium with either 100 or 500 µg/ mL ampicillin at 37 °C in 96-well plates in a microplate reader (Infinite M200pro multimode microplate reader, Tecan). The main culture (150 μL) was inoculated 1:1000 and growth curves recorded for 15 h. For better visualization, 1 divided by the time needed to reach an OD_600_ of 0.1 was defined as measure of the copy number.

### 4.6. Comparative Genomic Hybridization

Exponentially growing cells in LB (OD_600_ = 0.15) were mixed in a 1:1 ratio with cold killing buffer. Strain #1 (wt MG1655 with an inserted to site) grown in AB Glu-CAA until stationary phase was used as a reference. All samples were centrifuged at 4 °C and cells were resuspended in 300 µL immunoprecipitation buffer. Cells were sonicated via Bioruptor^®^ Plus (Diagenode Diagnosics) (48 cycles of 30 s with 30 s cooling) to receive DNA fragments of around 500 bp. The cell extract was centrifuged and the supernatant was transferred to a new reaction tube. TE buffer (300 µL) and 2 µL RNase A (10 mg/mL) were added, and samples were incubated at 65 °C for 90 min. DNA was extracted with phenol/chloroform. DNA (400 ng; 20 ng/mL in 20 µL) was labeled with Cy3-dCTP (sample) or Cy5-dCTP (reference), mixed and hybridized to whole genome microarrays from Agilent (8 × 15 k) as described [53]. Arrays were scanned on an Agilent SureScan High Resolution Scanner. Spot intensities were extracted via AgilentScan Control software. Ratios of dye intensities were calculated and normalized to the array-wide average using R. A Loess fitting was applied to the microarray data to obtain a locally weighted average (shown as the colored line in CGH plot). For this average line, we detected maxima and minima. The maximal and minimal positions were used to dissect the data set in subsets delimited by the extrema. For these subsets, a linear regression line was determined, and the coordinates of the intersection points were taken as final maxima and minima.

### 4.7. Flow Cytometry

To generate Rif/Ceph run out samples, cells were grown in LB-medium [with Ohly^®^ yeast extract] and treated with 150 µg/mL rifampicin and 10 µg/mL cephalexin in the early exponential phase for more than three generations (2–3 h), allowing them to finish ongoing rounds of replication [54]. For exponential phase samples, cells were grown in LB until the early exponential phase. The cells were harvested and washed twice in TBS (0.1 M Tris-HCl pH 7.5, 0.75 M NaCl). They were fixed in 100 µL TBS and 1 mL 77% ethanol and stored at least overnight at 4 °C. For run-out experiments, cell samples were washed and diluted in 0.1 M phosphate buffer, pH 9.0 (PB buffer). Proteins were stained overnight with 3 µg/mL FITC solution (PB buffer) at 4 °C. Afterwards, cells were stained with Hoechst 33,258 as has been outlined before [38]. They were analyzed on LSR II flow cytometer (BD Biosciences) and flow cytometry measurements were carried out as described [55]. *E. coli* MG1655 cells grown exponentially in AB-acetate and stained with Hoechst only served as an internal standard and were added to every sample. For DNA-stained exponential grown cells, the samples were washed in 0.5 M sodium-citrate and treated with 5 ng/mL RNase A in 0.5 M sodium-citrate for 4 h at 50 °C. They were stained with 250 nM SYTOX^®^ Green Nucleic Acid Stain (Thermo Fisher Scientific, Waltham, MA, USA ) and analyzed on Fortessa Flow Cytometer (BD Biosciences, San Jose, CA, USA). The SYTOX^®^ Green fluorescence was measured through a 530/30 nm bandpass filter. *E. coli* MG1655 cells grown exponentially in AB-acetate and stained with SYTOX^®^ Green served as standard. For protein-stained exponential grown cells, samples were treated with FITC as described above. The cells were washed with TBS and analyzed on Fortessa Flow Cytometer (BD Biosciences). The FITC fluorescence was measured through a 530/30 nm bandpass filter. Data was processed with the software FlowJo (Treestar, Ashland, OR, USA).

### 4.8. Fluorescence Microscopy and Data Evaluation

Cells were grown in AB glucose CAA to OD_450_ ~0.15. 1 mL of the culture was harvested by centrifugation and cells re-suspended in 25 µL fresh AB glucose CAA. Cells (2 µL) were transferred to 1% agarose pads containing 1% TAE. Fluorescence microscopy was performed with a Nikon Eclipse Ti-E microscope with a phase-contrast Plan Apo l oil objective (100; numerical aperture, 1.45) with the AHF HC filter set F36-528 YFP (excitation band pass [ex bp] 500/24-nm, beam splitter [bs] 515-nm, and emission [em] bp 535/30-nm filters) and an Argon Ion Laser (Melles Griot, Rochester, NY, USA). Images were acquired with an Andor iXon3 885 electron-multiplying charge-coupled device (EMCCD) camera. For quantification of fluorescence foci, 20 images were taken for every strain and the first 700 cells were used for further analyses. Images were analyzed by Fiji using the MircobeJ plugin [56].

### 4.9. Plasmid Construction

Plasmid pMA200 was constructed by the cutting of pMA308 with I-SceI and insertion of the *crtS* site amplified with primers 1593 and 1613 with *V. cholerae* chromosomal DNA as the template, by Gibson assembly [57]. Plasmid pMA308 was constructed by cutting pMA135 with AscI and inserting *oriC* amplified with primers 1349 and 1350 with *E. coli* MG1655 DNA as the template. Plasmid pMA899 was constructed by cutting of pMA135 with AscI and insertion of *oriF*, amplified with primers 1487 and 1488 with pMA129 as the template. Plasmid pMA206 was constructed by cutting of pMA899 with I-SceI and insertion of the *crtS* site, amplified with primers 1593 and 1613 with *V. cholerae* chromosomal DNA as the template, by Gibson assembly. Plasmid pMA568 was constructed by cutting pMA132 with I-SceI and insertion of a NotI restriction site flanked *lacZ* cassette amplified using primers 1002 and 1004. Plasmid pMA208 was constructed by a MoClo-reaction (with BsaI) with plasmid pMA350 and a PCR fragment amplified with primers 1661 and 1662 with *E. coli* DNA as the template. MoClo was carried out as described [27,58]. Plasmid pMA209 was constructed by a MoClo-reaction with plasmid pMA353 and a PCR fragment amplified with primers 1663 and 1664 with *E. coli* DNA as template. Plasmid pMA740 was constructed by a MoClo reaction with plasmid pMA351 and a PCR fragment amplified with primers 1204 and 1205 and plasmid pMA650 as the template to amplify an *ori2* without bpiI or BsaI restriction sites. Plasmid pMA734 was constructed by a MoClo reaction with plasmid pMA352 and a chloramphenicol resistance cassette flanked by FRT sites generated by PCR with primers 703 and 704. Plasmid pMA210 was constructed in a MoClo reaction (with bpiI) including plasmids pMA208, pMA209, pMA329, pMA734, pMA740 and pICH50927. Plasmid pMA157 was constructed by a MoClo reaction with plasmid pMA349 (with BsaI) and a PCR fragment amplified with primers 1439 and 1440 and *V. cholerae* chromosomal DNA as template. Plasmid pMA431 was constructed by a MoClo reaction (BsaI) with plasmid pMA350 and a kanamycin resistance cassette flanked by FRT sites generated by PCR with primers 1455 and 1456. Plasmid pMA710 was constructed as described for pMA711 before with pMA351 as MoClo vector instead pMA352 [27]. Plasmid pMA207 was constructed in a MoClo reaction (with bpiI) including plasmids pMA709, pMA710, pMA327, pMA431, pMA157 and pICH50900. Minichromosomes pMA108–116 were constructed by Gibson Assembly [57]. Inserts were amplified from pMA87 by PCR with primers that contain homologous sequences to sequences on pMA90, which contains only half of the *ori2* sequences, with the part missing starting close to the DnaA box. Primers used are given in supplementary Table S4. The vector pMA90 was linearized with NotI before assembly with the PCR products. Chemically competent *E. coli* DH5αλpir cells, which can replicate these minichromosomes using their R2K origin [59], were transformed with the Gibson Assembly products. Colonies were screened by colony PCR with primers 14 and 16. Primer 16 is located in a sequence missing in pMA90 but present in minichromosomes with a full *ori2*. All constructs were verified by sequencing. 

### 4.10. Strain Construction

Strain SM113 was constructed by introduction of the FROS array by P1 transduction and subsequent FRT recombination as described previously for strain SM112 [27]. To integrate *ori2* into *oriC* in *E. coli* we used a DNA fragment consisting of *ori2* and a chloramphenicol resistance cassette flanked by regions beside *oriC* (parts of *mioC* and *mnmG*) for homologous recombination (Figure 7). The subsequent DNA fragment was generated by releasing the insert of plasmid pMA210 by cutting with BsaI and transformed into strain AB330 for recombineering. Exchange of *oriC* for *ori2* was confirmed by PCR and flow cytometry and the respective strain designated NZ135. The *ori2* insertion was transferred to *E. coli* MG1655 wt by P1 transduction to give strain NZ134. Subsequently the CAT cassette was removed via pCP20-based recombination resulting in strain NZ138 [60]. Introduction of the *crtS* site into the chromosome was similar to *ori2* into *oriC* but using pMA207 instead of pMA210.

## Supplementary Materials

The following are available online at www.mdpi.com/2079-6382/7/1/3/s1, Table S1: Strains used in this study, Table S2: Plasmids used in this study, Table S3: Oligonucleotides used in this study, Table S4: Oligonucleotides used for construction of mutated *ori2*-minichromosomes.
