# Supplementary material for: Establishing a System for Testing Replication Inhibition of the Vibrio cholerae Secondary Chromosome in Escherichia coli
Source: Antibiotics (Basel). 2017 Dec 23;7(1):3. doi: 10.3390/antibiotics7010003 (PMC5872114; doi:10.3390/antibiotics7010003)
Supplement: Supplementary file 1 [file antibiotics-07-00003-s001.pdf]

Supplementary Materials: Establishing a System for Testing Replication Inhibition of the *Vibrio cholerae* Secondary Chromosome in *Escherichia coli*

Nadine Schallopp, Sarah Milbredt, Theodor Sperlea, Franziska S. Kemter, Matthias Bruhn, Daniel Schindler and Torsten Waldminghaus

Table S1. Strains used in this study.

Strain	Characteristics	Resistance	Reference
E. coli			
MG1555	wild type	-	[1]
AB330	*cf.* DY330, *lacZ+*, *gal+*	-	Alexander Böhm
XL1 Blue	*recA1 endA1 gyrA96 thi-1 hsdR17 supE44 relA1 lac*[F´ *proAB lacIqZ*ΔM15 *Tn10* (TetR)]	tetracycline, nalidixic acid	Stratagene
DH5αλpir	F- Φ80*lacZ*ΔM15 Δ(*lacZYA-argF*) U169 *recA1 endA1 hsdR17* (rK-, mK+) *phoA supE44 thi-1 gyrA96 relA1* λ+	nalidixic acid	[2]
WM3064	Donor strain for conjugation: *thrB1004 pro thi rpsL hsdS lacZ*ΔM15 RP4-1360 Δ(*araBAD*)*567* Δ*dapA1341*::[*erm pir*(wt)]	erythromycin	William Metcalf
#1	*MG1655 oriC*(wt), *tos1*	-	[3]
#15	*MG1655 oriC*(wt), *oriV*2, *tos1*	kanamycin	[3]
#16	*MG1655* Δ*oriC*(wt), *oriV*2, *tos1*	kanamycin	[3]
SMS18	MG1655 + pMA100	ampicilin	[4]
SMS29	MG1655 + pMA106	ampicilin	[4]
FSK18	MG1655 *parB(1070231-1070357) kanR*	kanamycin	[4]
SMS62	MG1655 + pMA129	ampicilin	[4]
NZ90	*cf.* #16; flipped KanR	-	this study
SM112	*cf.* #1; *lacZ::*TetO/LacO-array-FRT + pMA289	chloramphenicol	[5]
SM113	*cf.* #16; *lacZ::*TetO/LacO-array-FRT + pMA289	chloramphenicol, kanamycin	this study
NZ127	AB330 with *crtS* on chromosome	kanamycin	this study
NZ133	MG1655 with *crtS* on chromosome	kanamycin	this study
NZ139	MG1655 with *crtS* on chromosome, flipped KanR cassette	-	this study
NZ134	MG1655 Δ*oriC*::*oriII*	chloramphenicol	this study
NZ135	AB330 Δ*oriC*::*oriII*	chloramphenicol	this study
NZ138	MG1655 Δ*oriC*::*oriII*, flipped CmR cassette	-	this study
NZ140	NZ139 + pMA568	ampicilin	this study
NZ141	NZ139 + pMA899	ampicilin	this study
NZ72	MG1655 + pMA568	ampicilin	this study
NZ119	MG1655 + pMA899	ampicilin	this study
SMS169	WM3064 + pMA899	erythromycin, ampicilin	this study
NZ122	WM3064 + pMA206	erythromycin, ampicilin	this study
SM141	WM3064 + pMA308	erythromycin, ampicilin	this study
NZ115	WM3064 + pMA200	erythromycin, ampicilin	this study
V. cholerae			
El Tor N16961	wild type	streptomycin	[6]
MCH1	fused chromosomes lacking *oriII*	streptomycin	[7]
NSCV1	natural isolate with fused chromosomes	-	[8]

Table S2. Plasmids used in this study.

Plasmid	Characteristics	Resistance	Reference
pCP20	FLP+, λ cI857+, λ pR Rep^{ts}	ampicillin, chloramphenicol	[9]
pICH50927	Endlinker for MoClo reaction	ampicilin	[10]
pICH50900	Endlinker for MoClo reaction	ampicilin	[10]
pMA87	= synVicII-1.0	ampicilin	[4]
pMA90	= synVicII-0.2	ampicilin	[4]
pMA100	*oriII*-minichromosome (=synVicII-1.3)	ampicilin	[4]
pMA106	*oriC*-minichromosome(=synEsc-1.3)	ampicilin	[4]
pMA108	*oriII* minichromosome with scrambled DnaA box	ampicilin	this study
pMA109	*oriII* minichromosome with inverted DnaA box	ampicilin	this study
pMA110	*oriII* minichromosome with deleted DnaA box	ampicilin	this study
pMA111	*oriII* minichromosome with R3 DnaA box	ampicilin	this study
pMA112	*oriII* minichromosome with R2 DnaA box	ampicilin	this study
pMA113	*oriII* minichromosome with deletion left of DnaA box	ampicilin	this study
pMA114	*oriII* minichromosome with insertion right of DnaA box	ampicilin	this study
pMA115	*oriII* minichromosome with insertion left of DnaA box	ampicilin	this study
pMA116	*oriII* minichromosome with deletion right of DnaA box	ampicilin	this study
pMA129	replicon with oriF (=synF-plasmid)	ampicilin	[4]
pMA132	= synVicII-1.35	ampicilin	[11]
pMA135	=synVicII-1.351	ampicilin	[11]
pMA157	*crtS* for MoClo assembly	ampicilin	this study
pMA200	pMA308 with crtS	ampicilin	this study
pMA206	pMA899 with crtS	ampicilin	this study
pMA207	*crtS* insertion into *fucR-rlmM* region on *E. coli* chromosome	spectinomycin, kanamycin	this study
pMA208	*mnmG* flank for MoClo assembly	ampicilin	this study
pMA209	*mioC* flank for MoClo assembly	ampicilin	this study
pMA210	*oriII* insertion into *oriC* of *E. coli*	spectinomycin, chloramphenicol	this study
pMA289	pKG110 *tetR-mVenus*	chloramphenicol	[5]
pMA308	*oriC*-minichromosome with *oriT* for conjugation (longer region of oriC compared to pMA106)	ampicilin	this study
pMA327	MoClo backbone vector for pMA207	spectinomycin	[5]
pMA329	MoClo backbone vector for pMA210	spectinomycin	[5]
pMA349	MoClo backbone vector for *crtS*	ampicilin	[12]
pMA350	MoClo backbone vector for *mnmG* flank (for oriC to oriII exchange)	ampicilin	[12]
pMA351	MoClo backbone vector for *oriII* (for oriC to oriII exchange)	ampicilin	[12]
pMA352	MoClo backbone vector for CAT (for oriC to oriII exchange)	ampicilin	[12]
pMA353	MoClo backbone vector for *mioC* flank (for oriC to oriII exchange)	ampicilin	[12]
pMA431	kanamycin cassette for MoClo assembly	ampicilin, kanamycin	this study
pMA568	*oriII*-minichromosome (with NotI-flanked *lacZ*)	ampicilin	this study
pMA650	= synVicII-2.01	ampicilin	[11]
pMA709	*fucR-rlmM* 5′flank for MoClo assembly	ampicilin	[5]
pMA710	*fucR-rlmM* 3′ flank for MoClo assembly	ampicilin	[5]
pMA734	chloramphenicol cassette for MoClo assembly	ampicilin, chloramphenicol	this study
pMA740	*oriII* for MoClo assembly	ampicilin	this study
pMA899	*oriF*-replicon with *oriT* for conjugation	ampicilin	this study

Table S3. Oligonucleotides used in this study.

Name	Sequence (5′ → 3′)
14	CAATCTCAATTCGATCGGCCTGCACT
16	GGTTAGATCCGTATCACACTTACCGT
421	GTACGAGAGGAAGGAGATACAACGACGCCTATCTTGATGTCATCCACTCAGGTGTAAAGT TGACTGTGTGAGCACCTTG
422	GTACGAGAGGAAGGAGATACAACGACGCCTATCTTGATGTCATCCACTCAGGTAATAGGT GTACTGTGTGAGCACCTTG
423	GTACGAGAGGAAGGAGATACAACGACGCCTATCTTGATGTCATCCACTCAGGTCTGTGTG AGCACCTTGATC
444	GTACGAGAGGAAGGAGATACAACGACGCCTATCTTGATGTCATCCACTCAGGTTTTGGAT AAACTGTGTGAGCACCTTGATCATGC
474	GTACGAGAGGAAGGAGATACAACGACGCCTATCTTGATGTCATCCGGTTGTGGATAAACT G
475	GTACGAGAGGAAGGAGATACAACGACGCCTATCTTGATGTCATCCGACTTACTCAGGTTG TGG
476	GTACGAGAGGAAGGAGATACAACGACGCCTATCTTGATGTCATCCACTCAGGTTGTGGAT AAATGAGCACCTTGATC
477	GTACGAGAGGAAGGAGATACAACGACGCCTATCTTGATGTCATCCACTCAGGTTGTGGAT AAAGACTTCTGTGTGAGCACCTTGATC
478	GTACGAGAGGAAGGAGATACAACGACGCCTATCTTGATGTCATCCACTCAGGTTGTGTAT AAACTGTGTGAGCACCTTGATCATGC
498	GTACGAGAGGAAGGAGATACAACGACGCCTATCTTGATGTCATCCACTCAGGTTNNNNAT AAACTGTGTGAGCACCTTG
703	AAGGTCTCGGGAGGAAGTTCCTATACTTTCTAGAGAATAGGAACTTCGGAATAGGAACTT CATTTAAATGG
704	TTGGTCTCCAGCGAAGTTCCTATTCTCTAGAAAGTATAGGAACTTCGGCGCGCCTACCTGT GACGG
1002	TTGGTGAGAATCCAAGTAGGGATAATTGCGGCCGCAGCTGGCACGACAGGTTTGCCG
1004	AACTTCGCAGACCTATATTACCCTGATGCGGCCGCGACTATGCGGCATCAGAGC
1204	AAGGTCTCGGGAGCTAAAACGCACAAAGCCCG
1205	TTGGTCTCCAGCGGTAGGCAAAAAAAGAGCGAGC
1349	GAACTTCCTCGAGCATCTCCGACGGAAAGGTACCCGGTCCTTTGCTTGCGTTTAGTATC
1350	GAAAAATAAACAAATAGGGGTTCCGCGGGAAAGAATTCTAATCCCATACTTTTCCACAGG TAG
1439	AAGGTCTCGGGAGTGAGTGATCTTTTCACTATATTC
1440	TTGGTCTCCAGCGCTCTTTTAAGCAGGATCCTAAC
1455	AAGGTCTCGGGAGGAAGTTCCTATACTTTCTAGAGAATAGGAACTTCGGAATAGGAACTT CAAGATCCCC
1456	TTGGTCTCCAGCGAGTTCCTATTCCGAAGTTCCTATTCTCTAGAAAGTATAGGAACTTCAG AGCGCTTTTG
1487	GAGCATCTCCGACGGCGCGCCCATGGAGCGGCGTAACCGTC
1488	GGCCAGAGCTGCAGGCGCGCCCGTCGACAGCGACACACTTG
1593	TGGTGAGAATCCAAGTAGGGATAATGAGTGATCTTTTCACTATATTCTGTCTTAAGTCT
1613	AACTTCGCAGACCTATATTACCCTGTTATCTCTTTTAAGCAGGATCCTAACCGGA
1661	AAGGTCTCGGGAGGACCGCCGAGTGCATCCACTTC
1662	TTGGTCTCCAGCGCTTTTTAATACCCAGGATCC
1663	AAGGTCTCGGGAGATACTTATTTGAGTAAATTAAC
1664	TTGGTCTCCAGCGCTGGCTGGTTATCAGCTCCAC
3921366fw	GAGAATATGGCGTACCAGCA
3921366rv	AAGACGCAGGTATTTCGCTT
3921366p	CAACCTGACTTCGGTCCGCG 5′Fam-3′Tamra
ori2fw	CCTTGAGCTTGAGATTGCTG
ori2rv	GCCGCCCTACTATCGTTAAA
ori2probe	TGCTCACGCTGAGCCTCATTCA 5′Fam-3′Tamra

Table S4. Oligonucleotides used for construction of mutated *oriII*-minichromosomes.

Plasmid Name	Oligonucleotide Names
pMA108	421, 424
pMA109	422, 424
pMA110	423, 424
pMA111	444, 424
pMA112	478, 424
pMA113	474, 424
pMA114	477, 424
pMA115	475, 424
pMA116	476, 424

References

1. Blattner, F.R.; et al. The complete genome sequence of *Escherichia coli* K-12. *Science* **1997**, *277*, 1453–1462.
2. Miller, V.L.; Mekalanos, J.J. A novel suicide vector and its use in construction of insertion mutations: Osmoregulation of outer membrane proteins and virulence determinants in *Vibrio cholerae* requires *toxR*. *J. Bacteriol.* **1988**, *170*, 2575–2583.
3. Liang, X.; Baek, C.H.; Katzen, F. *Escherichia coli* with two linear chromosomes. *ACS Synth. Biol.* **2013**, *2*, 734–740.
4. Messerschmidt, S.J.; et al. Synthetic secondary chromosomes in *Escherichia coli* based on the replication origin of chromosome II in *Vibrio cholerae*. *Biotechnol. J.* **2015**, *10*, 302–314.
5. Schindler, D.; et al. Design and Assembly of DNA Sequence Libraries for Chromosomal Insertion in Bacteria Based on a Set of Modified MoClo Vectors. *ACS Synth. Biol.* **2016**, *5*, 1362–1368.
6. Heidelberg, J.F.; et al. DNA sequence of both chromosomes of the cholera pathogen *Vibrio cholerae. Nature* **2000**, *406*, 477–483.
7. Val, M.E.; et al. Genome engineering in *Vibrio cholerae*: a feasible approach to address biological issues. *PLoS Genet.* **2012**, *8*, e1002472.
8. Xie, G.; et al. Exception to the Rule: Genomic Characterization of Naturally Occurring Unusual Vibrio cholerae Strains with a Single Chromosome. *Int. J. Genomics*. **2017**, doi:10.1155/2017/8724304.
9. Cherepanov, P.P.; Wackernagel, W. Gene disruption in *Escherichia coli*: TcR and KmR cassettes with the option of Flp-catalyzed excision of the antibiotic-resistance determinant. *Gene* **1995**, *158*, 9–14.
10. Weber, E.; et al. A modular cloning system for standardized assembly of multigene constructs. *PLoS One* **2011**, *6*, e16765.
11. Messerschmidt, S.J.; et al. Optimization and characterization of the synthetic secondary chromosome synVicII in *Escherichia coli*. *Front Bioeng. Biotechnol.* **2016**, *4*, 96.
12. Zumkeller, C.M.; Schindler, D.; Waldminghaus, T. Modular assembly of synthetic secondary chromosomes. *Methods Mol. Bio*. **2017** (accepted for publication).

© 2017 by the authors; licensee MDPI, Basel, Switzerland. This article is an open access article distributed under the terms and conditions of the Creative Commons by Attribution (CC-BY) license (http://creativecommons.org/licenses/by/4.0/).
